# Society Meetings

**Published:** 1900-05

**Authors:** 


### SOCIETY MEETINGS.

The Buffalo Academy of Medicine held meetings during the month of April, 1900, as follows:

*Section on Surgery.*—Friday evening, April 6th. Program: Notes on Prostatectomy, Paul Thorndike, of Boston.

*Section on Medicine.*—Tuesday evening, April 10th. Program: Post chicken-pox nephritis, Dewitt H. Sherman; The treatment of scarlet fever, Henry R. Hopkins; Normal saline solution in gastro-enteric diseases of children, Irving M. Snow.

*Section on Ophthalmology, Otology, Rhinology and Laryngology.* – Monday, April 16th. Program: Some notes on absorption of cataract, Benjamin H. Grove. A. A. Hubbell and Benjamin F. Rogers led in the discussion which followed.

*Section on Pathology.*—Tuesday evening, April 17th. Program: Mistakes in gynecology and their pathological importance, Herman E. Hayd; discussed by M. A. Crockett and M. D. Mann.

*Section on Obstetrics.*—Tuesday evening, April 24th. Program: Limitations of gynecology, Prof. John B. Deaver, Philadelphia, Pa.

---

The next annual meeting of the fourth district branch of the New York State Medical Association, comprising fourteen counties of the western part of the state, will be held at the Buffalo Club, Delaware Avenue, this city, on Tuesday, May 8th, beginning at 10 o'clock a.m. Luncheon will be served at the Club by the Buffalo members. The meeting will be held under the presidency of Dr. Wm. H. Thornton, and an exceptionally large and interesting meeting is anticipated. Dr. E. D. Ferguson, of Troy, president of the State Medical Association; Dr. F. H. Wiggin, of New York, and other out-of-town guests are expected to be present and to take part in the proceedings.

At this writing the following papers have been promised: Hepatic sclerosis in nonalcoholics, A. L. Benedict; Title to be announced, Charles G. Stockton; Cases of injuries of the eye, Alvin A. Hubbell; Dislocation of tendons, and treatment, Wm. M. Bemus, Jamestown; Report of an epidemic of variola and varicella at Mount Morris during 1898, J. M. Hagey, Mount Morris; Treatment of typhoid fever, John H. Sackrider, East Randolph; Title to be announced, Elmer Starr; Hemorrhage after confinement and its treatment, Herman E. Hayd; A case of perforating gastric ulcer, Allen A. Jones; Goiter, E. J. Meyer; Intestinal adhesions, with report of cases, C. E. Congdon; Rupture of symphysis pubis during parturition, with report of a case, George A. Himmelsbach; Review of the present status of the operation of Jonnesco, Dr. M. Hartwig.

All physicians are invited to attend the meeting.

---

The National Confederation of State Medical Examining and Licensing Boards will hold its ninth annual meeting at Atlantic City, June 4, 1900. The Bulletin of the American Academy of Medicine, in its last issue speaks of this organization as follows:

"The meetings at Atlantic City are promising well; at least those which are the more closely associated with the *Bulletin*, for, of these only can we speak officially. Dr. Suiter, the Secretary of the National Confederation of State Medical Examining and Licensing Boards writes that preparations are making for their meeting, but it is too soon to make any definite announcement. The meeting will be held on Monday, June 4th, at a place to be determined upon later. This organisation has in it the potentiality for the good of the profession to a greater degree than almost any other combination of medical men. Hence there is a peculiar propriety in the imitation printed on the letter head: "Every state or territorial board whose duty is to examine or license physicians intending to practise in the jurisdiction of the board, by whatsoever name it may be called, is urged to affiliate with the National Confederation if it has not already done so.""

The present officers are: Dr. J. N. McCormick, Bowling Green, Ky., president; Drs. N. R. Coleman, Columbus, O., and B. F. Crummer, Omaha, Neb., vice-presidents; Dr. A. Walter Suiter, Herkimer, N.Y., secretary and treasurer; Drs. W. S. Foster, Pittsburg, Pa., J. M. Mathews, Louisville Ky., T. J. Happel, Trenton,

Tenn., William Warren Potter, Buffalo, N. Y., and Augustus Korndoerfer, Philadelphia, the executive council.

---

THE Niagara Falls Academy of Medicine will hold its annual meeting at the office of Dr. J. H. Miller, Monday, May 7, 1900, at 8.45 p.m.

The nominations for officers are as follows: President, Drs. Miller, Leo-Wolf, Scott, Guillemont; vice-president, Drs. Guillemont and McBrien; secretary and treasurer, Drs. Leo-Wolf, Miller, Potter; trustee vice Dr. Campbell, Drs. Scott and Campbell; council, Drs. Miller, McBrien and Potter.

Dr. Scott will deliver the presidential address. A full attendance is requested.

